# Revealing Maximal Diameter of Upper Limb Superficial Vein with an Elevated Environmental Temperature

**DOI:** 10.1155/2016/8096473

**Published:** 2016-08-15

**Authors:** Hira Irfan, Guo Shen Ooi, May M. Kyin, Pei Ho

**Affiliations:** ^1^Dow University of Health Sciences, Karachi 74200, Pakistan; ^2^Department of Cardiac, Thoracic and Vascular Surgery, National University Hospital, Singapore 119228

## Abstract

Ultrasonography is the primary tool for preoperative analysis of vein morphology for fistula creation in patients with end-stage renal disease. This study examines the effect of environmental temperature on the superficial vein size. Superficial veins of thirteen healthy volunteers were marked at three sites: cephalic vein in left lateral arm near cubital fossa, cephalic vein in left forearm at wrist, and basilic vein in left medial arm near cubital fossa. Mean diameters were recorded using ultrasound probe at 26°C and 43°C. Body temperature was increased using a Bair Hugger blanket. Mean values from the two temperatures were analyzed using paired sample *t*-test. All three superficial vein sites displayed statistically significant increase in diameter when the temperature was increased from 26°C to 43°C. Paired *t*-test showed *p* values of 0.001 for cephalic vein at wrist, 0.01 for cephalic vein near cubital fossa, and 0.01 for basilic vein near cubital fossa. This study proved that environmental temperature exerts a statistically significant effect on vein size measured by ultrasound during preoperative assessment for vascular access. Not to the extent of 43°C, the authors would recommend setting the room temperature higher during ultrasound vascular assessment to avoid underestimating the superficial vein size.

## 1. Introduction

According to a global overview by Grassmann et al. in 2005, more than 70% of end-stage renal disease (ESRD) patients were managed with hemodialysis [1]. Vascular access is an essential component of hemodialysis.

National Kidney Foundation (NKF) guidelines recommend duplex US as the essential study for upper arm vascular assessment before fistula creation [2]. The diameter of superficial vein was identified as a key predictor of access patency. Clinicians often make the decision of using native vein or artificial graft based on assessment of vein size by ultrasound [3].

The size of arteries remains relatively stable unless in extreme physiological conditions such as hypovolemic shock [4]. Veins, on the contrary, are capacitance vessels and their size may vary significantly with different physiological and environmental conditions. Underestimation of vein size may result in wasting patient's native vein suitable for fistula creation, whereas overestimation may result in a low maturation rate of the arteriovenous fistula (AVF) being created.

Numerous studies had explored thoroughly the potential of body positioning on vein size [5, 6]. However, not much work had investigated the effect of external environment. Our study aimed to assess variation in vein size in response to environmental temperature in a setting similar to regular vein size assessment performed for ESRD patient before fistula creation.

## 2. Materials and Methods

National Healthcare Group domain specific review board (NHG DSRB) approval was obtained for this project.

13 healthy adults were randomly selected to participate in this experimental study. All subjects were of average build and denied chronic ailments including diabetes, hypertension, and peripheral arterial disease. Research protocol was explained to all subjects in detail, their informed consent was obtained, and measures were taken to keep identity of all the participants strictly confidential.

Before initiating, participants were rested in a relatively stress-free environment for ten minutes. All measurements were obtained in a semirecumbent position on the left arm which was devoid of external pressures such as tight clothing or watches. Blood pressure measurements were taken in the right and the left arms and recorded. Three markings were made on the left upper limb: (i) lateral side of forearm 5 cm proximal to the styloid process of radius to mark cephalic vein in forearm (CV at wrist), (ii) lateral side of arm 5 cm proximal to the skin crease of cubital fossa to mark cephalic vein in arm (CV at cubital fossa), and (iii) medial side of arm 5 cm proximal to the skin crease of cubital fossa to mark basilic vein in arm (BV near cubital fossa). Internal diameter of veins was measured with the least pressure of ultrasound probe applied to these three sites. Readings were recorded to the accuracy of 0.01 cm using two-dimensional B mode ultrasound probe (SonoSite MicroMaxx) with an 11 MHz transducer. Average of the long and short axis of the ellipse was recorded at two separate occasions and the mean value was noted as the final diameter for each site.

The two temperatures under focus in the study were 26°C, which was the room temperature of the area used to conduct the experiment, and 43°C, which was selected to bring about a measurable change to prove our hypothesis. Measurements were first taken at 26°C and the participants were allowed to resume their normal activities for 10 minutes before making them rest in a Bair Hugger (neck to toe) for 10 minutes. Warm gel was applied and the measurements for the temperature of 43°C were recorded. One clinician carried out all measurements at 26°C and a different clinician recorded all readings at 43°C to achieve a balance between interobserver variation and observer bias.

SPSS STATISTICS 19 software for Windows was employed to analyze the data. Paired sample *t*-test was used to analyze if the differences in mean vein diameters at the two temperatures were obtained by chance alone. *p* value of <0.05 was considered significant, confidence interval was set at 95%, and quantile-quantile (Q-Q) plots were drawn to illustrate the normal distribution of data.

## 3. Results

Q-Q plots confirmed that the data was normally distributed. Blood pressure readings for all participants were within normal range.

**Figure 1:**
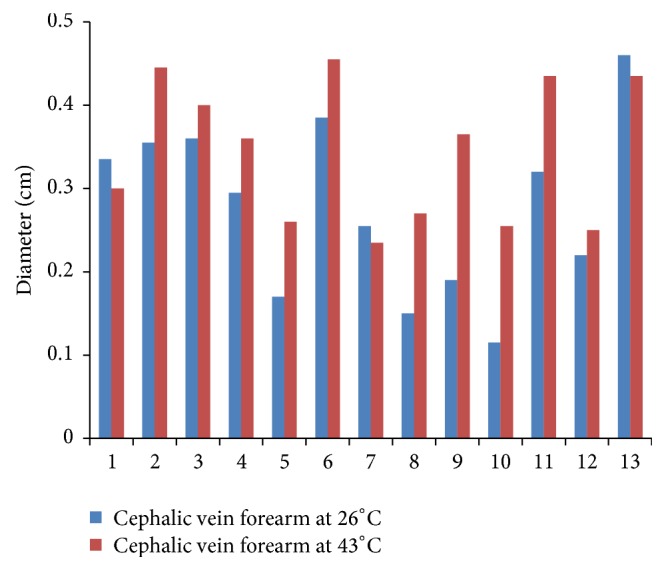
Diameter of cephalic vein at forearm.

**Figure 2:**
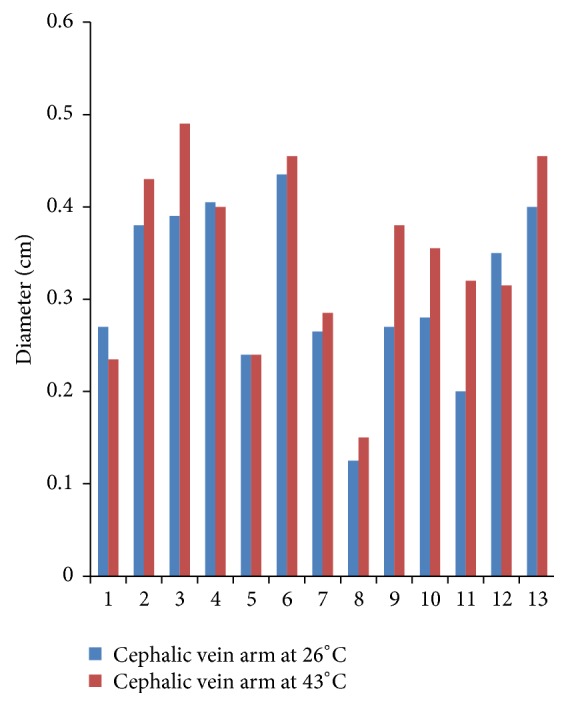
Diameter of cephalic vein at arm.

**Figure 3:**
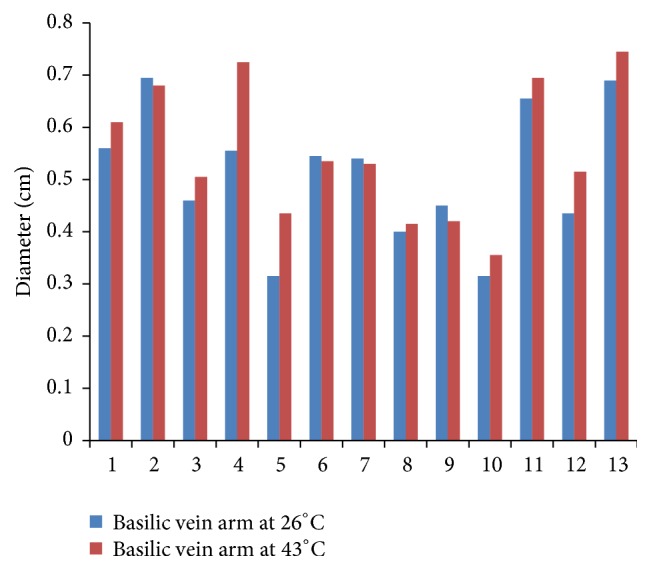
Diameter of basilic vein at arm.

Figures 1, 2, and 3 compare the diameters of veins at the three sites at 26°C and 43°C for all participants. Mean diameter of CV at wrist at 26°C was 0.28 cm (SD 0.10) which increased to 0.34 cm (SD 0.08) at 43°C. Mean diameter of CV near cubital fossa at 26°C was 0.31 cm (SD 0.09) which increased to 0.35 cm (SD 0.10) at 43°C. Mean diameter of BV near cubital fossa similarly increased from 0.51 cm (SD 0.13) at 26°C to 0.55 cm (SD 0.13) at 43°C. Paired *t*-test for mean diameters at the two temperatures showed *p* values of 0.001 for CV at wrist, 0.01 for CV near cubital fossa, and 0.01 for BV near cubital fossa. Hence, the increase in mean diameters of veins due to increase in temperature is not a result of chance alone and is statistically significant.

## 4. Discussion

Ultrasonography is the usual investigation for assessing superficial vein size prior to fistula procedures. Clinical examination of patient's arm and forearm with tourniquet on is useful in evaluating suitability of native vein for AVF creation. However, it could be difficult and misleading in certain patients. In an educational article, Brown commented on the beneficial impact of preoperative ultrasound vein size assessment for decision of vascular access creation [2]. Robbin et al. found that ultrasound vein mapping leads to a 31% increase in use of native vein for fistula creation compared with surgeons' decision of vascular access based on clinical examination only [7]. The NKF-KDOQI guideline suggested that the preferable mean venous diameter should be ≥2.5 mm and arterial diameter ≥1.6 mm for successful AVF creation [8]. Different from artery, vein size may vary tremendously in response to body posture, stress, and external environment [5, 6]. One would therefore prefer that ultrasound vein size assessment captures the maximum potential of the vein to facilitate clinical decision and, hence, better outcome [9]. Underestimation of vein size may result in wasting of patient's native vein good for fistula creation. Thus many researchers conducted studies to find out the optimal protocol for superficial vein size assessment. Van Bemmelen et al. and Korten et al. had examined the effect of body posture, tourniquet, and regional warm reagent around the upper limb on size of superficial vein [5, 6]. Their results state that warm water immersion of the limb and increased hydrostatic pressure during sitting increase venous diameter. Little attention has been paid to the external environment where the study is performed. Our study aimed to assess the vein size variation in response to environmental temperature in a setting similar to regular ultrasound vein size assessment performed for ESRD patients before fistula creation. In our study, an environmental temperature change affecting the whole body of the subjects was applied instead of regional temperature increase solely over the upper limb as this better mimics the actual environment of subjects. The result of this study showed that the cephalic and basilic vein size of majority of subjects increased at a higher environmental temperature as compared to their sizes at air-conditioned room temperature of 26°C. Currently, all the ultrasound vein size assessments for renal disease patients are carried out at a room temperature between 22°C and 26°C in our hospital. Underestimation of the size of the superficial vein is hence likely to exist in certain proportion of the patients. Not to the extent of 43°C, the authors would recommend setting the room temperature higher in the venue of ultrasound vascular assessment to avoid underestimating the superficial vein size. Alternatively, covering the patients with warm blanket or Bair Hugger before the ultrasound study would also help to reveal the optimal size of the superficial vein.

Healthy volunteers instead of renal disease patients were recruited for this study because the authors preferred to minimize any influence of medical comorbidities on the results. The study of Korten et al. showed that the change in vein size is comparable between healthy individual and dialysis patients towards various provocation methods [6]. Nonetheless, based on the result of the current study, a study with the same protocol should be performed on ESRD or renal impairment patients planned for vascular access surgery to confirm the response of their superficial vein towards environmental temperature.

The degree of change in vein size in response to environmental temperature represents the reactivity of a particular vein to external stimulation. It is uncertain whether the specific vein will show similar reactivity towards other stimuli such as arterializations, needling, or small size hematoma after AVF creation. If so, the reactivity of vein towards temperature change might help clinicians to predict the reactivity of AVF towards other stimuli. To understand more about the association, we need to conduct a study on the reactivity of the native superficial vein to temperature change and follow up the early and long term outcome of the AVF.

## 5. Conclusion 

Environmental temperature of the room in which vascular assessment is being conducted seems to be important in creating optimum conditions to facilitate clinical decision of vascular access creation. A similar study needs to be conducted on ESRD patients to further validate the clinical effectiveness of our results and set clearer guidelines.
